# Genetic Prognostic Factors in Multiple Sclerosis: Key Discoveries and Unmet Needs

**DOI:** 10.3390/ijms27083583

**Published:** 2026-04-17

**Authors:** Valentina Ciampana, Eleonora Virgilio, Loredana Paciolla, Sofia Asaro, Alessandro Franceschini, Muralidharan Thavamani, Letizia Mazzini, Cristoforo Comi, Nadia Barizzone, Sandra D’Alfonso, Domizia Vecchio

**Affiliations:** 1 Neurology Unit, Department of Translational Medicine, Maggiore Della Carità Hospital, University of Piemonte Orientale, 28100 Novara, Italy; 20022116@studenti.uniupo.it (V.C.); 20042425@studenti.uniupo.it (L.P.); sofia.asaro@maggioreosp.novara.it (S.A.); a.franceschini@maggioreosp.novara.it (A.F.); letizia.mazzini@uniupo.it (L.M.); domizia.vecchio@uniupo.it (D.V.); 2 Department of Clinical and Biological Sciences, San Luigi Gonzaga University Hospital, University of Turin, 10124 Torino, Italy; virgilioeleonora88@gmail.com; 3 Department of Health Sciences, Università del Piemonte Orientale (UPO), 28100 Novara, Italy; 20034829@studenti.uniupo.it (M.T.); nadia.barizzone@med.uniupo.it (N.B.); sandra.dalfonso@med.uniupo.it (S.D.)

**Keywords:** multiple sclerosis, genetics, genome-wide association studies (GWAS), personalized medicine, age at onset, relapse rate, cognitive, expanded disability status scale (EDSS), multiple sclerosis severity score (MSSS), cerebrospinal fluid

## Abstract

Multiple sclerosis (MS) is a chronic autoimmune and neurodegenerative disease characterized by marked clinical heterogeneity. While the genetic architecture underlying disease susceptibility is well established, the role of genetic factors in shaping disease prognosis remains clearly defined. In this structured narrative review, we examine available evidence on genetic contribution to key MS prognostic domains. This includes clinical outcomes, such as age at onset, relapse rate, disability progression, neurological sequelae, and cognitive impairment. We also consider radiological measures like brain and spinal cord lesion burden, gadolinium-enhancing lesions, and atrophy, as well as laboratory biomarkers, such as oligoclonal bands and Immunoglobulin G (IgG) index. Overall, current evidence suggests that genetic influences on prognosis are modest and highly heterogeneous. Only a limited number of associations—primarily from genome-wide association studies (GWAS)—have shown consistent replication, whereas many reported findings come from small candidate-gene studies and remain unconfirmed. Among these, the largest GWAS on age-related Multiple Sclerosis Severity Score (MSSS) identified a locus in the *DYSF–ZNF638* region reaching genome-wide significance. The strongest evidence from GWAS relates to relapse rate, magnetic resonance imaging (MRI) measures (e.g., thalamic atrophy) and intrathecal IgG synthesis, the latter also reaching genome-wide significance. Interpretation of genotype–phenotype associations is further limited by small sample sizes, limited replication, heterogeneity in study design with the predominance of candidate-gene approaches, variability in outcome definitions, treatment exposure, and population ancestry. These limitations currently preclude the routine use of genetic markers for prognostic stratification in clinical practice. Larger studies and collaborative genetic consortia efforts are needed to improve statistical power and reproducibility. Additionally, emerging epigenetic studies may provide valuable insights into prognosis and disease management. Understanding which genetic factors can predict diverse MS courses could enhance patient management and enable personalized treatment approaches.

## 1. Introduction

Multiple sclerosis (MS) is a chronic autoimmune and neurodegenerative disease characterized by marked clinical and radiological heterogeneity [1], making the prediction of disease course a major challenge in clinical practice. Its etiology is multifactorial, arising from the complex interplay between genetic susceptibility and environmental exposures. Despite major advances in understanding genetic susceptibility, the determinants of disease prognosis remain less clearly defined. This gap has important implications for risk stratification and personalized management in MS.

### 1.1. Role of Genetics in Aetiology: Current Evidence

Strong evidence of an inherited component in MS aetiology [2] has driven extensive efforts to identify the underlying genetic determinants. Current knowledge indicates that MS susceptibility is highly polygenic, with more than 230 common genetic variants contributing to disease risk. These include single nucleotide polymorphisms (SNPs) defined as genetic variants with a minor allele frequency greater than 1% within the major histocompatibility complex (MHC), human leukocyte antigen (HLA), non-HLA regions, and the X chromosome, collectively accounting for approximately 48% of heritability. The remaining heritability is likely explained by rare variants (minor allele frequency < 1%), epigenetics, and gene–gene (G-G) and gene–environment (G-E) interactions.

Early studies, guided by the “common disease, common variant” hypothesis [3], explained approximately 20% of MS heritability, with approximately 4% from the MHC region [4]. Familial aggregation studies further supported a genetic contribution. A recent study from our group [5] showed a higher mutational burden in multiplex MS families compared with sporadic cases and healthy controls (HCs), represented by both rare and common genetic variants. Large collaborative efforts, particularly the International Multiple Sclerosis Genetics Consortium (IMSGC), have been central in defining the genetic architecture of MS. Large cohorts of predominantly European ancestry were aggregated, with initial analyses focusing on 730 multiplex families, and more than 4000 SNPs were genotyped. Early analyses identified strong association signals within the MHC region, particularly the HLA-DRB1*15:01 haplotype, which remains the most robust genetic risk factor, with an odds ratio (OR) of approximately 3.6, without any dose effect [6]. Subsequent characterization of relevant variants within this region has been challenging, because of the high polymorphism of the region, with high gene density and linkage disequilibrium [4]. Until 2007, only HLA-DRB1*15, HLA-DQ*w6, HLA-A*03, and *HLA-B7* were consistently confirmed across multiple studies [7].

**Figure 1:**
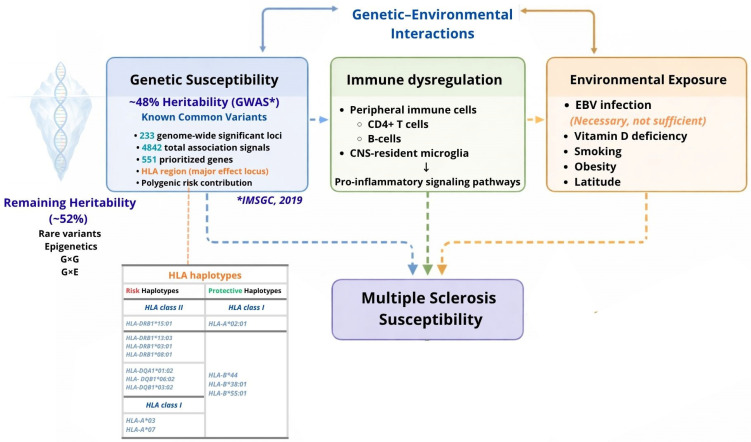
Genetic architecture is based on genome-wide association studies (GWAS*) data from the International Multiple Sclerosis Genetics Consortium (IMSGC, 2019 [9]), explaining ~48% of SNP-based heritability through common variants, including 233 genome-wide significant loci and 551 prioritized genes, with the HLA region as the major effect locus. Environmental exposures interact with genetic predisposition (G × E), contributing to immune dysregulation and MS development. The remaining heritability (~52%) may involve rare variants, epigenetic mechanisms, and gene–gene (G × G) interactions not fully captured by GWASs. * Data derived from the International Multiple Sclerosis Genetics Consortium (IMSGC), 2019.

The introduction of genome-wide association studies (GWASs), first introduced in 2007, marked a major shift toward hypothesis-free approaches, enabling the analysis of at least 600,000 SNPs, thereby identifying genetic susceptibility loci. Successive large-scale GWASs by the IMSGC, involving tens of thousands of patients and HCs, have progressively expanded the number of identified loci. The most recent study (47,000 patients and 68,000 HCs) tested over 8 million SNPs and identified 233 independent genome-wide significant associations, including 201 loci outside the HLA region and 32 additional signals within it, highlighting the complex and polygenic nature of MS susceptibility [8]. These associations meet the standard GWAS significance threshold (*p*-value < 5 × 10^−8^), as illustrated in Figure 1.

Beyond common variants, next-generation sequencing (NGS) approaches, including whole-exome, whole-genome and gene-panel sequencing, have explored the contribution of rare genetic variation. Although these studies suggest a potential role for rare variants, their contribution remains less well defined and requires further validation [10]. IMSGC also analyzed low-frequency and rare-coding variants on 68,379 MS patients and HCs using Exome Chip, a genotyping array containing over 100,000 rare non-synonymous variants and more than 2000 nonsense variants in the coding regions scattered across the genome. The study identified two novel genes driving MS risk independently of common-variant signals reported by the previous GWAS and estimated that up to 5% of this heritability is explained by low-frequency variation in gene coding sequence. Analyses of low-frequency and rare-coding variants indicate that rare variants may explain an additional proportion of heritability beyond common variants [9].

To capture the cumulative effect of multiple variants, genetic risk scores (GRSs), including unweighted (sum of the risk allele copies present in each human genome) and weighted approaches (wGRS, made by adjusting the risk allele by multiplying it by the natural logarithm of its weight), have been developed. While these improve the quantification of genetic susceptibility, they explain only part of disease risk and must be interpreted within the broader context of environmental exposures and lifestyle factors [11].

### 1.2. Known Prognostic Factors

The marked variability in prognosis can be attributed to multiple factors identifiable at onset. Negative prognostic factors can be broadly categorized into demographic, lifestyle, clinical, biological, and imaging domains. Demographic factors include male sex and age at onset (AAO), while lifestyle factors include smoking. Clinical predictors comprise motor, cerebellar, or sphincteric onset, higher annualized relapse rate (ARR), short interval between relapses, poor recovery after a relapse, higher Expanded Disability Status Scale (EDSS) at diagnosis, and the co-occurrence of other symptoms such as fatigue, cognitive decline, and depression. Biological factors include oligoclonal band (OCB) types II and III, increased intrathecal synthesis index markers, and elevated neurofilament light chain (NfL) levels in serum and cerebrospinal fluid (CSF), with possible contributions from tau and beta-amyloid proteins [12]. Imaging features with magnetic resonance imaging (MRI) include higher supratentorial lesion load, large or infratentorial lesions, cortical and spinal cord involvement, and brain and cervical atrophy [13]. Moreover, abnormal findings from ancillary tests, such as visual evoked potentials (VEPs), somatosensory evoked potentials (SSEPs), and optical coherence tomography (OCT), may further predict unfavorable outcomes. Despite the wide range of established clinical, laboratory, and imaging predictors, it remains unclear to what extent genetic background independently modulates these factors and, in turn, overall disease prognosis.

### 1.3. From Susceptibility Genetics to Prognostic Genetics

While the genetic architecture of MS susceptibility is now well characterized, translating these insights to disease course and long-term outcomes remains challenging. Prognostic outcomes likely reflect the cumulative effects of multiple small-effect variants acting through inflammatory activity, neurodegeneration, and repair mechanisms and may be further modified by exposure to disease-modifying treatments (DMTs) and environmental factors.

The hypothesis that shared genetic risk factors could modulate disease severity, convincingly demonstrated only for the HLA-DR*15 allele, remains debated [14]. Consistent with this, Harding et al. found no associations between 10 non-HLA SNPs and time to disability milestones, AAO, or time to secondary progression (SP) [15] in a cohort of over 1000 patients followed for 14.1 years.

Overall, these findings suggest that genetic determinants of prognosis may be partly distinct from susceptibility loci and/or that prognostic effects are small and difficult to detect without large, well-phenotyped longitudinal cohorts.

### 1.4. Aims

**Figure 2:**
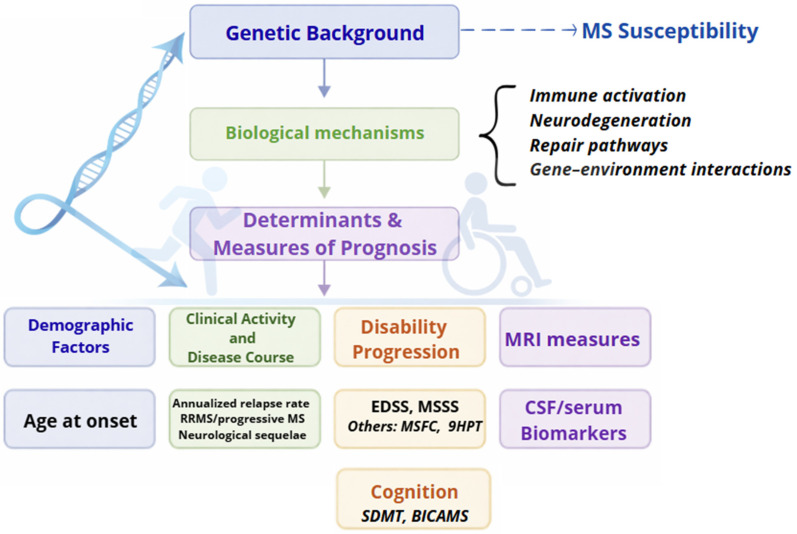
Conceptual framework linking genetic background to prognostic domains in multiple sclerosis. Genetic background may influence prognosis through pathways involving immune activation, neurodegeneration, repair mechanisms, and gene–environment interactions, contributing to variability across key domains (age at onset, clinical activity, disability progression, cognition, imaging, and fluid biomarkers). Representative but non-exhaustive examples are shown. This framework is conceptual and hypothesis-generating and does not imply causal relationships. Abbreviations: EDSS, Expanded Disability Status Scale; MSSS, multiple sclerosis severity score; 9HPT, 9-hole peg test; MSFC, MS Functional Composite; SDMT, Symbol Digit Modalities Test; BICAMS, Brief International Cognitive Assessment for MS; MRI, magnetic resonance imaging; CSF, cerebrospinal fluid.

This review specifically addresses the role of genetic factors in shaping disease course in MS. We provide an integrated and critical synthesis of genetic associations across prognostic domains, explicitly evaluating the strength, consistency, and limitations of the available evidence. In particular, we consider evidence derived from HLA variants [16], SNPs in candidate-gene studies [17], and genome-wide approaches, distinguishing between replicated findings and preliminary associations. We aim to offer a more nuanced and clinically relevant interpretation of prognostic genetics in MS. However, the field remains rapidly evolving [18]. A conceptual framework summarizing these relationships is presented in Figure 2.

## 2. Methods

We performed a structured narrative review. The marked heterogeneity in study design, genetic methodologies, outcome definitions and follow-up duration across available studies precluded a formal systematic review or meta-analysis. Therefore, this work provides a structured qualitative synthesis aimed at maximizing thematic coverage.

We focused on studies investigating associations between genetics and the most widely used predictors of disease course across clinical, imaging, and laboratory domains; for the first one, we included age at onset, type of onset (symptoms at onset, relapse rate, neurological sequelae), disability progression, and cognition.

A literature search was conducted in PubMed, Scopus and Embase. We included English-language studies published between January 2000 and May 2025. Search terms included combinations of the following keywords: (“multiple sclerosis” or “MS”) AND (“genetics”, “gene”, “GWAS”, “polygenic risk score”) AND (“prognosis”, “disease progression”, “disability”, “age at onset”, “EDSS”, “MSSS”, “disease severity”, “annual relapse rate”, “relapse rate”, “sequelae”, “relapsing–remitting”, “progressive MS”, “conversion to SPMS”, “cognitive”, “cognition”, “MRI”, “atrophy”, “lesions”, “OCT”, “VEP”, “SSEP”, “CSF”, “kappa index” or “K index”).

Studies were selected based on their relevance to genetic associations with disease prognosis. The initial search yielded approximately 2900 records. Inclusion criteria were original human studies investigating associations between genetic variants and clinical outcomes or disease progression in MS. Screening was performed by title and abstract, followed by full-text evaluation. Exclusion criteria included lack of prognostic outcomes, non-original data, inadequate reporting of genetic methods, or non-MS population. Moreover, we excluded case reports, case series, in vivo studies, and non-human studies. When potential overlap between cohorts was suspected, priority was given to the most comprehensive or recent analyses to minimize redundancy. Study selection and data extraction were independently performed by two authors; disagreements were resolved by discussion. Finally, we considered relevant for qualitative discussion approximately 300 publications. When multiple studies showed similar associations, priority was given to larger cohorts, replicated studies, and genome-wide approaches over small candidate-gene analyses, although such designs remain relatively scarce in this field. Given the heterogeneity of the available evidence, findings were interpreted with caution, with particular emphasis on study design, sample size, and replication status.

Tables were constructed to facilitate comparison across studies and highlight methodological differences and consistency of findings.

## 3. Age at Onset

AAO shows substantial variability in MS, ranging from pediatric-onset MS (POMS, <18 years) to adult-onset (AOMS) and late-onset MS (LOMS, >50 years). LOMS is generally associated with a distinct clinical trajectory, including more frequent motor involvement; faster disability accumulation, even if influenced by the male gender and spinal cord onset; an earlier conversion to SPMS; and a higher frequency of other autoimmune diseases [19,20,21,22,23]. Although AOMS and LOMS show different courses, some authors believe that the same risk factors in AOMS were probably responsible also for LOMS, with a different AAO influenced by the exposure to environmental factors [23]. In a Scandinavian study regarding co-affected sib-pairs, AAO and disease course emerged as under genetic control, with *HLA-DR2* carriers having an earlier onset [24]. In contrast, the genetic load of familiar cases compared to sporadic MS was not related to AAO, even if in affected relative pairs, the AAO was proportional to the genetic sharing [25]. The role of genetic burden in predicting AAO was then sustained by epidemiology studies; in the African-American MS population, the disease is more severe, with older AAO and clinical manifestations restricted to the optic nerves and spinal cord compared to the White population [26].

From a genetic perspective, evidence linking specific variants to AAO remains variable and depends on the methodological approach.

The most consistent signal derives from the HLA region in pioneer studies. In particular, the HLA-DRB1*15:01 has been associated with a modest but reproducible reduction in AAO across multiple cohorts, possibly with a maternally transmitted haplotype [27]. SNPs in the promoter of the HLA-G gene or in tight LD [28] and carriers of the HLA-DRB1*03 haplotype have been associated with lower AAO [29]. However, the magnitude of this effect is limited, and not all studies confirmed a clear relationship between HLA genetic burden and AAO [30].

Polygenic approaches provide additional insights. A wGRS including 107 MS susceptibility variants showed divergent effects across disease phenotypes: in bout-onset MS, a higher wGRS was associated with earlier AAO, whereas in PPMS it was associated with delayed onset [31]. These findings suggest that susceptibility variants may preferentially influence the timing of the inflammatory phase rather than the progressive phase of the disease. Consistently, a comprehensive investigation of MS-risk variants in 3495 patients (both HLA-DRB1* 15:01 and a GRS accounting for 198 SNPs outside HLA-loci) regarding AAO showed an accelerated onset in those with a higher MS-genetic risk burden [32].

To date, no genetic associations with AAO have yet reached robust and consistently replicated genome-wide significance. Most available evidence derives from HLA-focused analyses or polygenic approaches based on previously identified susceptibility variants, rather than from dedicated GWASs of AAO.

Beyond these approaches, multiple candidate-gene studies have reported associations between individual variants outside the HLA region and AAO (Table 1).

Overall, Table 1 highlights the heterogeneity of findings across non-HLA loci and the lack of consistent replication, limiting their current interpretability. Importantly, even studies targeting the same gene may substantially differ in study design, population characteristics, and outcome definitions (e.g., early-onset vs. continuous AAO, as for Δ32 deletion in *CCR5*), leading to apparently inconsistent findings [33,35].

Despite these limitations, several variants have been proposed as potential modifiers of AAO. Variants in osteopontin (*OPN*), a pleiotropic protein over-transcripted in MS [34]; alphaB-crystallin, a molecular chaperone and myelin antigen [36]; peroxisome proliferator-activated receptors (*PPAR-gamma* and *PPAR-alpha*), crucial in regulating autoimmunity [37]; and adhesion molecules such as intercellular adhesion molecule 1 (*ICAM-1*), involved in immune cell trafficking across the blood–brain barrier (BBB) [41], have been reported to be associated with earlier AAO in individual studies. Variants in the *ABCB1* gene, which affects P-glycoprotein function and molecular transport across the BBB, have also been suggested to modulate AAO [44].

However, these findings remain inconsistent, and several susceptibility variants, including ones in the cytotoxic T lymphocyte-associated antigen-4 (*CTLA4*) [45] and in the tumor necrosis factor receptor superfamily member 1A (*TNFRSF1A*) gene [46], have not shown clear associations with AAO.

Additional findings involving specific pathways further illustrate the complexity of genetic influences on AAO. For example, carriers of the T-allele in the methylenetetrahydrofolate reductase (*MTHFR*) gene, whose protein is associated with serum homocysteine levels, have been associated with an earlier disease onset compared to other genotypes [38]. In contrast, genetic predictors of low vitamin D levels have not shown a consistent association with AAO, which seems to independently correlate with HLA-DRB1*1501 [47].

A more recent area of investigation concerns the genetic determinants of LOMS. A population-based study using Swedish nationwide registries found no significant difference in MS risk among individuals with relatives diagnosed with AOMS versus LOMS [48]. Future studies are needed to identify possible genetic variants specifically associated with LOMS [49].

Overall, genetic factors appear to have a modest influence on AAO. The strongest support comes from HLA-DRB1*15:01 and polygenic approaches, whereas most non-HLA candidate-gene associations remain preliminary. Heterogeneity of study design, limited statistical power, and inconsistent replication across cohorts remain the main challenges in defining the genetic determinants of AAO.

## 4. Clinical Features

### 4.1. Type of Onset

There is limited evidence regarding correlations between type of onset and genetic loci. After searching PubMed using the following keywords (“multiple sclerosis” AND (visual OR ambulation OR sensory OR motor OR sphincteric OR brainstem OR type of onset) AND (gene OR genetics OR genetic loci)), only a few studies were found. Kulakova et al. found a possible association between type onset and the rs1800693 SNP in 508 MS patients [47]. *SIRT1* gene polymorphisms (rs3758391 and rs7895833) may be related to optic neuritis in a small case–control study (N = 79), with or without a following MS diagnosis, even if not influencing SIRT1 blood levels [48].

Finally, sensory symptoms seem to be rarer in women with MS with the TT-genotype in the rs1801157 SNP in *CXCL12* (N = 250 MS; *p* = 0.004); interestingly, this genotype was also associated with a shorter disease duration (*p* = 0.07) [49].

### 4.2. Annualized Relapse Rate

Recent evidence has explored the association between genetic variants and ARR, a key measure of inflammatory disease activity in MS.

Early studies have mainly focused on the HLA region, with inconsistent findings. HLA-DRB1*15:01 was not associated with ARR or disability progression in cohort analyses [50], whereas the HLA-DRB1*03 allele has been linked to higher relapse rates in smaller studies [29]. Moreover, HLA-DRB1*15:01 may exert an indirect effect by modulating the relationship between vitamin D levels and ARR, although this has been observed only in POMS [51]. Similarly, GRS and ancestry-related analyses have not shown a clear association with ARR in available cohorts (N = 181) [51].

Beyond classical HLA-DRB1 effects, additional variants within the HLA region (e.g., rs9266773 in HLA-B*44:02, rs9277561, and rs927756) have been associated with ARR [50]. Outside the HLA region, several candidate-gene studies have reported associations between individual SNPs and relapse activity. The risk genotype (CT + TT) at rs12959006 in myelin basic protein (*MBP*) has been associated with increased hazard of relapse (*p* = 0.005) [52], with subsequent confirmation in an independent cohort (*p* = 7 × 10^−5^) [53].

More robust evidence comes from genome-wide approaches, although results remain limited. In a multi-cohort longitudinal GWAS (N = 449) from Australia and the USA, a SNP in the low-density lipoprotein-related protein 2 (*LRP2*) gene (rs12988804) was the first polymorphism reported to reach genome-wide significant association with ARR (*p* = 3.30 × 10^−8^) [54], and this finding was subsequently replicated in an independent cohort (N = 527 Belgian MS patients with 970 documented relapses) with nominal statistical significance [55]. This represents one of the strongest pieces of evidence for a genetic contribution to relapse activity to date.

Additional associations have been reported in smaller cohorts. In a POMS population (N = 102), four SNPs (rs11154801, rs650258, rs12212193, rs2303759) were associated with higher ARR; one variant (rs11154801) was confirmed in an AOMS cohort (N = 141) [56]. Similarly, in an Iranian study (N = 102), a SNP (rs12722489) in *IL-2RA* was associated with ARR [57].

More recently, a Belgian GWAS for ARR in the first two years after diagnosis and prior to initiation of DMT (discovery + replication: N = 991, total = 2.231 relapses) showed an association with a rare variant in the 3’-UTR region of the *WNT9B* gene, not related to MSSS [58]. Although biologically plausible given the role of WNT signaling in CNS development and repair, this finding has not yet been independently replicated. Finally, variants in the vitamin D receptor (*VDR*) gene have also been explored. In particular, the CC genotype and C allele have been associated with higher ARR in the first year (*p* = 0.006), whereas the CT genotype was associated with lower ARR (*p* = 0.009; N = 80) [59].

Overall, genetic influences on ARR appear modest. Only a limited number of associations have reached genome-wide significance, with even fewer being independently replicated (e.g., *LRP2*, whereas evidence for *WNT9B* remains unreplicated). Most available evidence derives from candidate-gene studies with modest sample sizes. In addition, the interpretation of genetic associations with ARR is complicated by several factors, including differences in follow-up duration and the confounding effects of DMTs, which limit comparability across studies.

### 4.3. Neurological Sequelae

Neurological sequelae, defined as incomplete or partial recovery from a clinical attack, represent an important contributor to long-term disability accumulation in MS [16]. To date, genetic evidence in this domain remains extremely limited. An Italian group (N = 400) reported that incomplete recovery after relapse, a key determinant of neurological sequelae, was associated with an increased frequency of the rs9897526 A-allele of the granulin precursor gene (*GRN*, OR 4.367, *p* = 0.005) [60].

Moreover, SNPs rs7975232, rs1544410, rs731236, and rs2228570 of *VDR* have been associated with rehabilitation outcomes, assessed by EDSS and pain numerical rating scores at the beginning and the end of the rehabilitation treatment [61]. While these findings suggest a potential role of genetic factors in recovery and functional outcomes, they derive from small, context-specific studies and require further validation.

### 4.4. Relapsing–Remitting or Progressive Course

The genetic contribution to clinical course—particularly the transition from RRMS to progressive forms (SPMS or PPMS)—remains incompletely understood, with overall limited and heterogeneous evidence.

Early studies have mainly focused on the HLA region. The HLA-A*02:01 allele, known to be protective for MS susceptibility, has been associated with a reduced risk of conversion to SPMS [32]. In addition, Dcunha et al. reported that HLA-DRB1*01 was more frequent in SPMS compared to RRMS, whereas HLA-DRB1*03 (*p* = 0.03) and HLA-DRB1*15:01 (*p* = 0.02) were associated with RRMS, even after adjustment for OCB status [29].

Beyond the HLA region, most available evidence derives from hypothesis-driven candidate-gene case–control studies. Variants in adhesion molecules, such as E-selectin, have been associated with RRMS, whereas specific genotypes appeared less frequent in patients progressing to SPMS [62]. Similarly, variants in apoptosis-related genes (e.g., rs2037815 GG genotype in *CASP8*) have been associated with PPMS [63]. Variants related to oxidative stress pathways have also been implicated: two SNPs in the cytochrome b-245 alpha chain (*CYBA*) gene (rs1049254/G and rs4673/A) were associated with reduced reactive oxygen species production and a delayed progression to SP (>20 years, *p* = 0.003) [64]. Variant rs1544410 in the *VDR* gene was associated with shorter time to progression (*p* = 0.046) [59]. Additional studies investigated genes involved in immune regulation and neuroinflammation: Wagner et al. found no association between polymorphisms in *CD28, CTLA-4, CD80* and *CD86*, and AAO and duration of the relapsing–remitting phase (*p* = 0.18) [40]. However, findings across candidate-gene approaches remain inconsistent, often derived from small cohorts, and generally lack independent replication.

More recently, through exon sequencing and genotyping, several MS-susceptibility SNPs have been associated with clinical course: *CACNA1H* has been linked to RR-onset, whereas variants in the NLR family pyrin domain containing 5 (*NLRP5*) [65] and in the progranulin (*GRN*) gene have been associated with PPMS. In *GRN*, the rs2879096 TT-genotype and rs4792938 C allele were associated with PPMS (N = 354 PPMS versus 343 HC; *p* = 0.023 and *p* = 0.011, respectively) [66]. However, these findings remain preliminary and have not been consistently replicated.

To date, GWASs have not identified robust and consistently replicated loci associated with PPMS; however, various genetic variants in the field of oxidative stress and immune dysfunction appeared promising [67].

Taken together, current evidence suggests that genetic factors may contribute to MS’s clinical course, but their effects are likely modest and context dependent. The lack of large, well-powered longitudinal studies and the heterogeneity in phenotype classification and evolving disease definitions under updated diagnostic criteria [68,69] remain major barriers to identifying reliable genetic predictors of disease progression.

## 5. Disability Progression

### 5.1. How to Measure Disability in Genetic Studies?

Disability in MS is most commonly assessed using the EDSS, a 10-point ordinal scale with half-point increments derived from ratings across several neurological domains, including visual, brainstem, motor, sensory, cerebellar, bladder/bowel, and cognitive function, corrected for walking distance [70]. Although EDSS is widely used in association studies, its application in genetic studies has important limitations, as disability progression typically requires longitudinal assessment over at least two time points and often several years (minimum five) of follow-up. The MSSS was developed to contextualize EDSS by correcting for disease duration and is expressed as a decile rank from 0 to 10 [71]. As it is calculated on a single EDSS assessment, it shares the same limitations, including operator dependency, being strongly influenced by ambulation, and being insufficient for predicting long-term progression. The progression index (PI, calculated from the EDSS divided by duration) is an alternative continuous variable. Importantly, these metrics may not adequately capture silent progression, such as progression independent of relapses (PIRA), which remains challenging to define. Newer, more efficient, and precise disability scales have been developed, such as the Multiple Sclerosis Functional Composite (MSFC), a scale also administered by non-medical personnel. The MSFC includes the 9-hole peg test (9HPT) for upper limb function, the Timed 25-Foot Walking Test for walking ability, and the 3 s Paced Auditory Serial Addition Test for cognitive abilities.

These methodological limitations should be taken into account when interpreting genetic studies of disability.

### 5.2. Evidence of a Link Between Genetic and MS Disability

The relationship between genetic background and disability progression remains incompletely understood, with overall limited and heterogeneous evidence.

Both single variants and cumulative approaches based on genetic risk scores (GRS/wGRS) have been investigated. Several studies suggest that susceptibility-related variants and severity-related variants may be only partially overlapping, implying that the biological processes underlying disease risk and long-term disability may differ [72]. Large-scale studies have generally yielded negative or modest results. In 2010, Jensen CJ et al. found no significant associations between a cumulative GRS based on susceptibility loci and EDSS, MSSS, or AAO (N = 1006 MS), although weak trends for isolated SNPs were observed [14]. In 2011, a GWAS by the IMSGC, including more than 2.5 million SNPs (N = 1470 MS), did not identify loci associated with MSSS at genome-wide significance [73]. In a larger cohort (N > 7000 MS), neither GRS nor wGRS was associated with MSSS after adjustment for cohort, sex, AAO, and HLA-DRB1*15:01 [74]. Taken together, these findings suggest that MS susceptibility burden alone has limited value for disability severity.

Consistently, studies investigating classical susceptibility loci have not demonstrated a clear role in disability progression. For example, the association between apolipoprotein E (*APOE*) polymorphisms and MS severity has been extensively debated. A large study by Masterman T. et al., including over 900 MS patients, found no differences in genotype or phenotype frequencies between benign and severe disease forms (EDSS ≤ 3 within 10 years of onset and EDSS > 6 within 8 years) [45], as supported in other studies [75,76,77]. Although smaller studies suggested potential interactions with environmental factors, such as smoking in *APOE-E4* carriers [78], larger studies did not support these findings. Similarly, Javor J. et al. failed to demonstrate a role for the susceptibility risk factor tumor necrosis factor (*TNF*) in relation to disability outcomes [46]. Moreover, 10 non-HLA MS-associated SNPs in a cohort of over 1000 patients followed for 14 years showed that none was associated with time to key disability milestones (EDSS 4, 6, or 8) [15].

More recent studies have explored alternative analytical strategies. A novel machine-learning approach based on longitudinal EDSS trajectories in 269 MS cases (2786 longitudinal EDSS assessments) [79] identified seven loci, out of known MS-susceptibility loci, associated with worsening disability over time. Likewise, Jokubaitis VG et al. suggested that clinical outcomes may be associated with multiple loci of small effect sizes in more than 1800 RRMS and showed that combining genetic background with clinical variables at diagnosis may improve prognostic stratification. They predicted MS severity using a machine learning approach (over 62.000 SNPs), confirming that clinical heterogeneity was unrelated to genetic risk factors [72]. However, these approaches remain difficult to compare directly across studies and still require external validation.

In contrast, some studies suggest that susceptibility variants may contribute, at least in part, to disability outcomes. Pan G et al. showed that 116 MS-associated SNPs predict conversion from clinically isolated syndrome (CIS) to MS, ARR, and EDSS over five years in 127 patients with a first demyelinating event, supporting a possible polygenic effect on early disease course [18].

Within this framework, the role of the HLA region—despite being the strongest genetic determinant of susceptibility—remains uncertain in relation to disability progression. HLA-DRB1*08 has been associated with a longer time to reach EDSS 6.0, whereas HLA-DRB1*01 was linked to a shorter time to this milestone in a Lithuanian cohort (N = 120 MS) [80]. In an Iranian cohort, carriers of the HLA-DRB1*15:01/15:01 and those with combined HLA-DRB1*15:01 and *CD24* risk genotypes showed higher MSSS values [81]. Other studies suggested potentially protective effects for HLA-A*02 and unfavorable effects for HLA-B*07, HLA-B*44, HLA-B*08, and HLA-DQB1*06 [82]. The same group reported inconclusive results regarding HLA-DRB1*15:01. Another study demonstrated that the cumulative HLA genetic burden (HLA-GB) was not related to MSSS or conversion from CIS to MS; only HLA -DRB1*08 was found to be associated with lower disability and conversion to SPMS [30].

Several studies have also investigated genetic factors associated with disability progression (EDSS and MSSS), largely independent of MS susceptibility loci (Table 2).

Most studies summarized in Table 2 are hypothesis-driven candidate-gene association analyses, often limited by small sample sizes and lack of replication. Only a minority employed broader approaches, including multi-gene panels or GWAS-informed designs, and few included independent replication cohorts. Heterogeneity in outcome definitions (e.g., EDSS, MSSS, PI, or categorical severity measures) further limits comparability. As a result, these findings should be considered preliminary.

Nevertheless, a few studies have provided relatively more robust signals compared to the rest of the literature. For example, a multi-cohort analysis identified variants in the *MGAT5* locus associated with MSSS, with *p*-values approaching genome-wide significance; however, the strongest signals were located in intergenic regions, limiting biological interpretability [86]. In line with these results, some authors suggested the hypothesis that severity may depend on complex genetic architectures, including gene–gene interactions, rather than single variants.

Consistently, larger and more comprehensive approaches have not supported a major role for individual variants. A study by Kreft KL et al. evaluating progression-associated SNPs identified in GWAS analyses (N= 1455 MS) did not support the clinical utility of individual genotyping [102].

The role of rare variants has also been explored using exome sequencing approaches. A discovery cohort of 20 MS patients (10 with benign and 10 with aggressive disease course), followed by validation in two independent cohorts (N = 194 MS: 107 benign, 87 aggressive, and N = 257 MS: 224 benign, 33 aggressive), revealed two polymorphisms, rs28469012 in *CPXM2* (carboxypeptidase X, M14 family, member 2) and rs10894768 in *IGSF9B* (immunoglobulin superfamily member 9B) [49]. Functional analyses supported their potential biological relevance: in chronic active MS lesions, *IGSF9B* was expressed in astrocytes and macrophages/microglial cells, whereas *CPXM2* and *NLRP9* expression was restricted to macrophages/microglia.

### 5.3. Beyond EDSS and MSSS: Emerging Approaches to Disability Progression

Beyond traditional EDSS- and MSSS-based approaches, recent studies have explored alternative and more refined measures of disability progression, providing additional insights into the genetic architecture of MS severity.

A large GWAS on age-related MSSS, including 12,584 cases, identified a locus in *DYSF-ZNF638* associated with a 3.7-year earlier need for walking assistance in homozygous carriers, along with increased brain pathology. Another locus in the DNM3-PIGC region showed significant heritability enrichment in CNS tissues, highlighting the potential role of neurodegeneration, CNS resilience and neurocognitive reserve in shaping MS outcomes [103].

Emerging approaches integrating metabolic and functional pathways have also shown promising, although still preliminary, results. Genetic scores based on serine metabolism have been associated with an increased risk of disability worsening, suggesting a potential role for metabolic pathways in MS progression [104]. Similarly, the Brain-Derived Neurotrophic Factor (*BDNF*) Val66Met (rs6265) polymorphism has been associated with a greater improvement in walking function following rehabilitation (*p* = 0.024) in progressive MS, measured with the six-minute walk test, without significant association for the 10-metre walk test and 9HPT for manual dexterity.

## 6. Cognitive Measures

### 6.1. How to Measure Cognitive Outcomes?

Cognitive impairment (CI) affects up to 40–60% of MS [105], regardless of clinical course and disease evolution. The pattern includes attention span deficits, slowing in processing speed, executive and memory dysfunctions, manifesting independently of motor disability progression, and making early detection challenging [106].

Cognition should be assessed in an early baseline screening with the Symbol Digit Modalities Test (SDMT) [107]. In addition, other scales grouped into neuropsychological test batteries have been validated for MS patients, and the most used are Rao’s Brief Repeatable Battery (BRB) and the Brief International Cognitive Assessment for MS (BICAMS) [108]. Cognitive performance has been previously associated with EDSS, cortical lesions and brain atrophy on MRI, but the exact mechanisms underlying cognitive dysfunctions are still under debate [109].

### 6.2. Evidence of a Link Between Genetics and Cognition

Genetic studies investigating cognitive outcomes in MS are relatively limited and have primarily focused on candidate genes. Firstly, the *APOE* genotype has been related to cognitive status. In fact, *APOE-E4* carriers are more likely to have at least one impaired cognitive test [110] and, in a systematic review and meta-analysis (13 studies, five quantitative analyses), a more delayed stimulus response and visuospatial impairment [111].

Another extensively studied candidate gene is *BDNF*, which plays a key role in synaptic plasticity and neuroprotection [112]. In 2011, the risk allele rs2030324 has been associated with a protective effect against visual cognitive processing deficits, as measured by SDMT, and with preserved left thalamic volume (N = 209) [113]. Another polymorphism (rs6265 Val66Met) has been linked to cortical thickness at diagnosis, a structural correlate predictive of cognitive impairment [114]. Conversely, a case–control study in a Mexican cohort reported that the Met allele was associated with an increased risk of cognitive impairment, potentially reflecting reduced BDNF-mediated neuroplasticity [115]. However, the connection between BDNF and CI remains controversial, as not all studies have demonstrated a consistent association [116]; in addition, some authors have highlighted the influence of confounding factors such as depression [117].

More recently, variants outside classical neurotrophic pathways have been explored. The *LRP2* risk allele (rs12988804) has been associated with decreased performance on the California Verbal Learning Test-II in a PPMS cohort of 60 patients, also characterized for environmental and cardiovascular risk factors [118]; findings have not been replicated.

Overall, available data suggest a potential genetic contribution to cognitive impairment in MS; however, the evidence remains limited and often inconsistent, with few replicated findings and a predominance of small candidate-gene studies. Notably, no large GWASs specifically addressing CI in MS are currently available, despite the relatively higher reproducibility of cognitive measures compared to other clinical outcomes, highlighting a significant gap in the current literature.

## 7. Imaging Features

### 7.1. Magnetic Resonance Imaging

Genetic association studies using MRI-derived outcomes have provided promising insights, in particular regarding measures of neurodegeneration and structural brain changes.

Early studies identified associations between genetic variants and MRI measures. The *BDNF* rs6265 SNP (Val66Met) was associated with higher grey matter (GM) volume, particularly in the cingulate cortex [112,119], and a lower T2 lesion volume [112]; subsequent studies suggested a potential protective effect on brain atrophy [120]. Another study reported that heterozygosity for rs2227139 within the MHC class II region was associated with right frontal periventricular lesion distribution [121]. In addition, variants in genes belonging to the histone deacetylase superfamily have been linked to differences in brain volume measures [122]. Moreover, a SNP (*rs1818879)* in the *IL6* gene may be associated with a higher prevalence of gadolinium-enhancing lesions at the time of diagnosis [123]. A meta-analysis for disease progression, measured by whole brain and T2-lesion volume, showed that some SNPs in non-risk loci, such as the neurodevelopmental protein tyrosine phosphatase receptor type D *(PTPRD)* gene and the ubiquitin ligase (*NEDD4L*) gene, were related to progression [124]. Additionally, cerebral atrophy at onset was more frequent in carriers of the CC genotype and C allele of rs1544410 in *VDR* (*p* = 0.04) [59]. Recently, following the identification of rs10191329 as the first locus for MS severity, it has been investigated if it was associated with MRI measures in a discovery (N = 748 RRMS) and a replication cohort (N = 360 RRMS) showing an association with 28% greater brain atrophy for carriers [59].

The contribution of the HLA region to MRI phenotypes has also been investigated, with inconsistent results.

A monocentric Italian cohort investigated the role of HLA-GB in cross-sectional volumetric measures of white matter (WM), GM, hippocampus and thalamus, and the number of T2-hyperintense lesions, finding a correlation with hippocampal volume and thalamic volume but not with T2-lesion volumes (N = ~200) [125]. Similarly, HLA-genetic burden has been associated with reduced WM fraction in female RRMS [30]. Some other studies reported associations with HLA-DRB1*15:01 and radiological measures (lesion load, atrophy), but not *HLA-A02:01* [126]; however, they appear to not be replicated [127]. HLA-DRB1*15:01 carriers showed a greater annualized change in T2-lesion volume (*p*< 0.025), a higher number of gadolinium-enhancing lesions, and a faster rate of spinal cord atrophy (*p*< 0.05), in line with previous studies [128].

Following the identification of MS susceptibility loci through a GWAS, polygenic approaches have been applied to imaging phenotypes. Genetic risk scores (GRS and wGRS) have been associated with thalamic atrophy within 10 years of disease progression (*p* < 0.001). Twelve SNPs from the GWAS [129] were associated with mean upper cervical cord area (N = 141 MS), with three of them protective; the cumulative wGRS showed the same trend [130]. However, other GWAS-based analyses did not identify associations with cortical thickness, even if a trend was detected in gene sets involved in glutamate signaling, neural development and an adjustment of intracellular calcium concentration [131]. Shams et al. developed a GRS made by the largest available MS GWAS dataset (N = 41.505 MS), which was validated in independent UK Biobank and Northern California cohorts, demonstrating an association between genetic susceptibility and thalamic atrophy within 10 years of disease [132].

More recent studies have adopted pathway-based approaches. A candidate-pathway analysis showed that the sodium homeostasis gene pathway was associated with GM volume in PMS, whereas the inflammatory pathway and its regulation were associated with T2-lesion volume in RRMS. At a single-variant level, T-allele carriers in rs7104613 of *SPON1* were associated with reduced deep GM volume in PMS, whereas carriers of the A-allele in rs740948 of *SEMA3A* were associated with WM volume in RRMS [133]. Variants in immune-related pathways have also been implicated. Coding variants in complement component 3 (C3) (rs2230199, rs11569415) have been associated with global and local brain atrophy, particularly in subcortical structures such as the thalamus, amygdala, striatum, and hippocampus, in a large multi-center cohort [134].

In recent years, increasing attention has been directed toward fluid and imaging biomarkers as complementary and potentially more sensitive measures of disease activity, particularly in clinical trials and therapeutic monitoring. In this framework, the concept of No Evidence of Disease Activity (NEDA-3)—absence of clinical relapses, disability progression, and new or enlarging MRI lesions—has emerged as a composite outcome integrating clinical and radiological measures [135]. Preliminary genetic data in this field remain limited; however, specific variants such as the CC genotype of rs2069812 in the *IL5* gene have been associated with achieving NEDA-3 status over a three-year follow-up (*p* = 0.007), suggesting a potential genetic contribution to composite disease control (*p* = 0.007) [136].

Overall, beyond the established role of HLA-DRB1*15:01 in determining brain atrophy, only a few well-powered approaches, including large GWAS-based analyses and candidate-pathways analyses, provided robust insights, but no variants have consistently reached genome-wide significance for MRI measures of disease severity. However, despite being often underpowered and poorly replicated, MRI-based outcomes are more objective and standardized than clinical measures, making them promising intermediate phenotypes for genetic studies of MS severity.

### 7.2. Optical Coherence Tomography

OCT is used to predict clinical and imaging-based outcomes. The thickness of the inner retinal layer has proven to be an indirect surrogate for optic nerve involvement and has been correlated with known genetic factors in preliminary studies. A GWAS of the structural and functional visual pathway in MS, replicated in an independent cohort (overall N > 300–500 per analysis), identified several genetic predictors of ganglion cell/inner plexiform layer atrophy, with the most consistent one represented by the early complement *C3* gene, *C1Qa*, and *CR1* (*p* values in the 10^−4^ range) [137]. Despite OCT being a less widely used and largely ancillary tool in MS assessment, these findings are notable as they derive from a GWAS with independent replication, in contrast to the more limited and less consistent genetic evidence available for MRI-derived outcomes.

## 8. Blood and Cerebrospinal Fluid Biomarkers

The production of CNS-auto-reactive antibodies, predominantly immunoglobulin G (IgG) class, evaluated by OCB types II and III, represents an immunological hallmark of disease; OCB+ is observed in 90–95% of MS patients in Northern Europe. Increased IgG index and kappa index are quantitative surrogates of dissemination in time in the newest revision of McDonald’s criteria [69,138].

OCB+ is believed to be influenced by genetic factors: CSF abnormalities are found in 19% of unaffected siblings, as opposed to 4% of unrelated HCs [139], and they vary according to ethnicity [140].

Only a few studies investigated the role of genetics in predicting CSF features. Early candidate-gene studies identified associations within the HLA region: HLA-DRB1*15:01 was associated with OCB+, whereas HLA-DRB1*04:04 was associated with OCB- in an Italian cohort [16]; these findings were partially replicated in a Scandinavian and a Lithuanian cohort, where HLA-DRB1*15:01 was associated with OCB+, whereas HLA-DRB1*08 was associated with lower IgG index values [80].

More robust evidence derives from genome-wide approaches. In 2015, the largest and most methodologically robust GWAS to date (N = 3026 MS in the discovery and N = 3891 in the replication phase), across nine countries, provides strong evidence for a genetic contribution to intrathecal IgG synthesis. Haplotypes tagging HLA-DRB1*15:01 and HLA-DQA1*03:01 were strongly associated with OCB status (*p* = 8.88 × 10^−16^). In addition, a novel locus near the *ELAC1/SMAD4* genes (rs9807334) was identified (*p*~10^−7^). For IgG index, the strongest association signal was observed at the immunoglobulin heavy chain locus (*p* = 3.79 × 10^−37^), together with independent effects within the MHC region [141]. These findings, derived from a large, multi-cohort design with replication, provide some of the most compelling evidence supporting a genetic influence on CSF humoral immune response in MS.

Consistently, after a GWAS on the IgG index, five SNPs with genome-wide significance were detected and replicated, all located around the immunoglobulin heavy chain locus on chromosome 14 and in linkage disequilibrium; the top signal was in an intronic region of the IG gamma3 heavy chain gene. They also identified an association between the GM21* haplotype and a higher IgG index [142].

In 2025, a large multicenter collaborative GWAS, including 3934 MS patients in the discovery cohort and 1094 in the replication cohort, identified a novel genome-wide significant association between an intronic variant in the *SAMD5* gene (rs844586; *p* = 1.48 × 10^−8^) and intrathecal IgG synthesis. This signal was shown to be independent of the previously described MHC association. The study also suggested that genetic susceptibility to MS, measured through PRS, could be associated with both the presence and the magnitude of intrathecal IgG synthesis, further supporting a genetic contribution to the humoral immune response in MS [143].

Beyond intrathecal antibody production, a parallel series of studies [144,145,146] was conducted to explore CSF protein biomarkers linked to MS genetic variants. A key finding was that CSF levels of the kinesin superfamily proteins, primarily *KIF5A*, were associated with common susceptibility genotypes (rs12368653 and rs703842), suggesting their potential as biomarkers of disease progression.

Finally, recent work has linked genetic variants associated with MS progression [89] to markers of neuroaxonal damage expressed by serum NfL. In a longitudinal cohort (N = 658) prospectively monitored every 2 years for less than a decade, the rs10191329 risk variant found in a previous GWAS was not associated with baseline serum NfL levels, indicating similar neuroaxonal damage at diagnosis. However, in homozygous carriers, they found higher serum NfL levels in follow-ups, preceding increased disability progression and suggesting a delayed effect on neurodegeneration [147].

Overall, these findings support a genetic contribution to intrathecal immune activity and neuroaxonal damage in MS, with the strongest evidence derived from a large GWAS including multi-cohort designs and replication analyses. In light of the new diagnostic criteria for MS [138], further investigation is warranted not only for the IgG index, but also for KFLC and K index.

## 9. Discussion

MS is influenced by a wide spectrum of both common and rare polymorphic variants. While most variants exert modest individual effects (except for HLAB1*15:01), their combined contribution, together with gene–gene and gene–environment interactions, substantially contributes to disease susceptibility. According to IMSGC (2019) [9], SNP-based heritability explains approximately 48% of MS heritability, whereas the remaining proportion is likely attributable to rare variants, epigenetic mechanisms, and additional gene–gene and gene–environment interactions.

While this genetic architecture of risk is now well established, the genetic determinants of disease course appear to be modest, heterogeneous, and only partially overlapping with risk loci. Notably, genetic associations appear more robust when considering intermediate phenotypes, such as imaging and CSF biomarkers, rather than conventional clinical outcomes. In this review, we summarize the evidence on genetic associations with prognosis across clinical, biological, and imaging markers. The main added value of this work lies in the integration of genetic associations across these multiple prognostic domains, typically investigated separately in the literature.

### 9.1. Evolution of Prognostic Genetic Studies

Early prognostic studies were largely based on the hypothesis that the same susceptibility variants could also predict disease course. For example, Kalincik et al. evaluated associations between sixteen genetic susceptibility markers and MS phenotypes in terms of clinical features (conversion to MS, ARR, disability) and imaging (whole and partial brain volumes, lesion load and volumes) but found no significant associations [148]. Moreover, Lin R. et al. demonstrated that five SNPs in known risk loci could be associated with relapses and levels of vitamin D, enlightening both the prognostic role of genetic risk loci and the interaction between genetics and environmental risk factors [149].

Over time, research has shifted toward identifying dedicated prognostic genetic factors through hypothesis-free approaches such as GWASs. In parallel, the subsequent revisions of McDonald’s criteria and the major changes in using MRI and CSF data for diagnosis have altered patient selection and disease definition, further contributing to variability across studies.

### 9.2. Why Are Prognostic Genetic Associations Still Limited?

Despite the recent advances, robust genetic predictors of MS prognosis remain limited. A major challenge is the modest effect size of individual variants, which requires large and well-characterized populations to achieve adequate statistical power. While international consortia have enabled genome-wide studies [132], only a few prognostic associations have reached genome-wide significance (*p*-value threshold of 5 × 10^−8^), such as variants in *LRP2* related to relapse rate [54] and in *SAMD5* related to intrathecal IgG synthesis [143]. Most findings from candidate-gene studies have not been replicated yet and should be interpreted with caution.

Moreover, substantial heterogeneity across studies further limits comparability. Clinical outcomes are inconsistently defined across studies, with variability in endpoints such as EDSS, MSSS, relapse rate, and imaging measures. Differences in follow-up duration, data collection methods, and cut-off definitions limit comparability and reduce the feasibility of meta-analyses. Moreover, the predominance of European ancestry cohorts restricts generalizability to other populations. As a consequence, only a few meta-analyses were available on limited features and putative genes.

Finally, interpreting prognostic genetic studies in modern cohorts is challenging due to the widespread use of DMTs. Nowadays, treatments substantially alter the natural history of MS, making it difficult to identify patient populations in which the independent contribution of genetic factors to disability progression can be reliably assessed. As a consequence, traditional disability outcomes such as EDSS progression may be strongly influenced by the treatment exposure rather than by the underlying biological course of the disease. In addition, commonly used disability measures such as EDSS have intrinsic limitations, including non-linear structure and strong dependence on ambulatory function.

**Figure 3:**
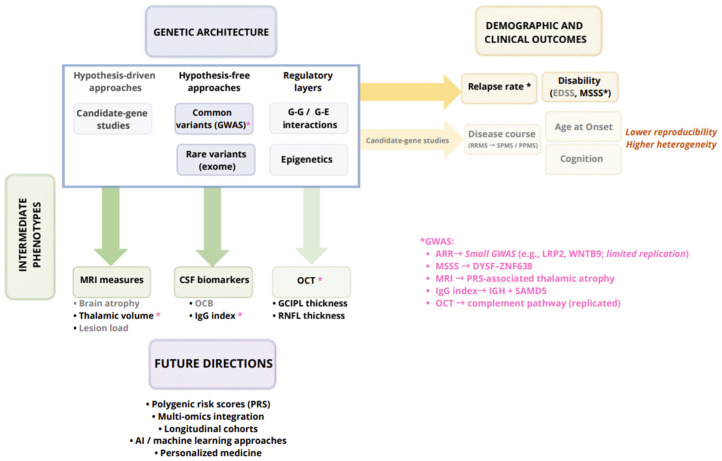
Genetic evidence across outcome domains in multiple sclerosis. Schematic overview of genetic approaches and key outcome domains in multiple sclerosis (MS) prognosis. Hypothesis-driven (candidate-gene) and hypothesis-free approaches (genome-wide association studies (GWASs), exome sequencing), together with regulatory mechanisms (gene–gene (G–G) and gene–environment (G–E) interactions and epigenetics), have been used to investigate genetic associations with clinical and demographic outcomes, including relapse rate, disability (Expanded Disability Status Scale (EDSS), multiple sclerosis severity score (MSSS)), disease course, and cognition, as well as intermediate phenotypes, such as magnetic resonance imaging (MRI) measures, cerebrospinal fluid (CSF) biomarkers (oligoclonal bands (OCBs), immunoglobulin G (IgG) index), and optical coherence tomography (OCT) parameters (retinal nerve fiber layer (RNFL), ganglion cell/inner plexiform layer (GCIPL)). Representative GWAS signals include *DYSF ZNF638* for MSSS, polygenic risk score (PRS)-associated thalamic atrophy for MRI, *IGH* and *SAMD5* loci for CSF IgG synthesis, and complement pathway genes for OCT. For ARR, preliminary associations have been reported (e.g., *LRP2*, *WNTB9*), although these derive from relatively small cohorts. Future directions are shown below. Dark arrows indicate more robust and reproducible associations, whereas light arrows indicate weaker or less consistent evidence.

In this context, genetic studies focusing on early disease features and intermediate phenotypes—such as imaging measures and fluid biomarkers—may provide a more informative approach to understanding the biological pathways underlying disease progression. Genome-wide studies have identified limited but robust associations with disability severity, particularly for MSSS, where large-scale analyses have revealed reproducible loci (e.g., *DYSF–ZNF638* [103]). By contrast, GWASs on relapse-related outcomes have generally been conducted in smaller cohorts and yielded less consistent findings [54,55,58]. A consistent pattern across studies is that genetic associations appear more robust for intermediate phenotypes such as MRI and CSF measures, whereas conventional clinical negative prognostic factors show weaker and less reproducible associations. This is particularly evident for MRI- and CSF-derived measures: polygenic susceptibility has been associated with longitudinal thalamic atrophy [132], whereas large GWASs have identified robust associations with intrathecal IgG synthesis [143]. Emerging evidence from an OCT-based GWAS further supports this concept, highlighting complement pathway genes as contributors to structural and functional visual pathway damage [137]. These intermediate phenotypes may also be less affected by clinical and treatment-related confounders, providing a more direct readout of underlying disease biology. By contrast, analogous evidence for conventional disability outcomes remains weaker and far less consistent. Overall, the available evidence suggests that genetic factors contribute to multiple dimensions of MS prognosis, but their effects are modest and context dependent. The most robust signals derive from large genome-wide and replicated studies, while the majority of candidate-gene associations remain preliminary. Finally, while genetic predictors of MS prognosis remain limited, integrating genetic data with clinical, imaging, and fluid biomarkers represents a promising strategy toward personalized medicine in MS. A schematic summary of the genetic architecture underlying MS prognosis, integrating study approaches and outcome domains, is presented in Figure 3.

### 9.3. Future Perspectives

Nonetheless, further prognostic data could come from epigenetic studies, as demonstrated by methylation studies and studies on long non-coding RNAs [150].

A further interesting field of investigation is the genetic influence on brain pathology. As an example, a study [151] analyzed 179 MS brain donors from the Netherlands Brain Bank MS autopsy cohort that were genotyped for 102 SNPs, selected based on their reported associations with clinical outcome or their associations with genes that show differential gene expression in MS lesions. Three SNPs linked to MS clinical severity showed a significant association between the genotype and either the proportion of active lesions (rs2234978/*FAS* and rs11957313/*KCNIP1*) or the proportion of mixed active/inactive lesions (rs8056098/*CLEC16A*). Three SNPs linked to MS pathology-associated genes showed a significant association with either the proportion of active lesions (rs3130253 in the myelin oligodendrocyte glycoprotein gene), the incidence of cortical gray matter lesions (rs1064395 in *NCAN*), or the proportion of remyelinated lesions (rs5742909 in *CTLA4*). This field of investigation is interesting since it translates genotypes linked to clinical MS outcomes into mechanisms involved in the lesion pathogenesis.

Understanding which genetic factors can predict different disease courses not only offers insights into the fundamental etiology of MS but also provides a better framework for patient management at the time of diagnosis, refining prognostication and enabling the development of tailored therapies.

## 10. Conclusions

We present the current state of the art on the influence of genetics on the most common outcomes. Future goals will be the development of prognostic scores, grouping genetic variants associated with major clinical, blood and CSF and imaging biomarkers, to better define MS patients’ prognosis at the time of diagnosis and better direct the choice among different DMTs, as well as allow a tailored therapy.

## Figures and Tables

**Table 1:** Timeline of genetic loci associated with age at onset (AAO).

Variant and/or Locus	Gene/Region	Cohort	Outcome	Study Design	Statistical Significance *p*-Value	Replication Status	References
32-bp deletion (*CCR5* Δ32)	*CCR5*	219 *HLA-DR4*+ MS	Negative association with early AAO	Candidate-gene association study	*p* = 0.0115	Not replicated	Favorova OO, et al., 2002 [33]
3 SNPs (8090th, 9250th, and 9583rd position)	*OPN*	116 MS/124 HC	Delayed AAO (protective genotype in 9583rd polymorphism)	Candidate-gene (case–control) association study	*p* = 0.01	Not replicated	Niino M, et al., 2003 [34]
32-bp deletion (*CCR5* Δ32)	*CCR5*	331 RRMS and SPMS/ 108 PPMS/ 230 HC	Earlier AAO in RRMS/SPMS; PPMS: no correlation	Candidate-gene (case–control) association study	*p* = 0.003	Not replicated	Silversides JA et al., 2004 [35]
Multiple SNPs in the promoter and intronic region	*CRYAB*	233 MS/ 96 HC	Earlier AAO for g.*CRYAB*-249GG genotype	Candidate- gene (case–control) association study	*p* = 0.05	Not replicated	Stoevring B et al., 2006 [36]
Leu162Val and Pro12Ala	*PPARA* and *PPARG*	116 MS/ 211 HC	Delayed AAO for Pro12Ala	Candidate- gene (case–control) association study	*p* = 0.006	Not replicated	Klotz L. et al., 2008 [37]
C677T polymorphism	*MTHFR*	194 MS/ 230 HC	Earlier AAO for T-allele	Candidate- gene (case–control) association study	*p* = 0.04	Not replicated	Alatab S. et al., 2011 [38]
54 SNPs	*TRAIL*, *TRAILR-1*, *TRAILR-2*, *TRAILR-3* and *TRAILR-4*	509 MS treated with IFN-beta + 226 MS (replication cohort)	rs1047275 in *TRAILR-2* (1) and rs7011559 in *TRAILR-4* (2) promising in the discovery cohort, not replicated	Candidate- gene (case–control) association study + replication cohort	*p* (discovery/replication cohort/joint analysis), (1) = 0.009/0.363/>0.1; (2) = 0.012/0.252/0.009	Failed in replication	López-Gómez C. et al., 2013 [39]
11 polymorphisms	*CD28, CD80, CD86*	336 MS/ 322 HC	Association in combined models	Candidate- gene (case–control) association study	At least one minor allele in each of the three loci, *p* = 0.27; 1 SNP in CD80 and CD86, *p* = 0.049; Receptor–ligand interactions, *p* = 0.002	Inconclusive	Wagner M. et al., 2015 [40]
2 SNPs: rs1799969 (G241R), rs5498 (K469E)	*ICAM-1*	248 MS/ 208 HC	Early AAO with G/G at rs5498	Candidate- gene (case–control) association study	*p* = 0.04	Not replicated	Shawkatová I. et al. 2017 [41]
147 SNPs	9 adhesion and trafficking genes * in lymphocyte	389 MS/ 336 HC	Dose dependent effect for rs1250249 of *FN1*	Candidate- gene (case–control) association study	*p =* 0.0002	Not replicated	Dardiotis E. et al., 2017 [42]
38 SNPs	21 genes (*PSMG4,* *NLRP5,* *MC1R*)	100 MS (discovery) ** + 2016 MS (replication)	Earlier AAO for *PSMG4* (p.W99R), and *NLRP5* (p.R761L); delayed AAO for *MC1R* (p.R160W)	Candidate- gene association study + replication cohort	*p* = 0.010, *p* = 0.041	Inconclusive (partial replication)	Sadovnick AD et al., 2017 [25]
Minor G-allele in SNP rs948854	Galanin	110 MS	Association with severity/AAO context	Candidate- gene association study	*p* = 0.004	Not replicated	Lioudyno V et al., 2019 [43]
C1236T, G2677T/A, and C3435T	*ABCB1*	199 MS/ 200 HC	Modulation of AAO for G2677 allele	Candidate- gene (case–control) association study	*p* = 0.0001	Not replicated	Guerrero Camacho JL et al., 2023 [44]

* P-selectin (*SELP*), integrins (*ITGA4, ITGB1*, and *ITGB7*), adhesion molecules (*ICAM1, VCAM1*, and *MADCAM1*), fibronectin 1 (*FN1*), and osteopontin (*SPP1*); ** The primary analysis of the discovery cohort nominated 38 variants in 21 genes; a candidate-gene approach for the replication cohort was done.

**Table 2:** Timeline of genetic loci associated with Expanded Disability Status Scale (EDSS), multiple sclerosis severity score (MSSS), and related disability outcomes.

Type of Variant and Locus	Gene/Region	Cohort Size	Outcome	Study Design	Statistical Significance	Replication Status	References
Multiple SNPs	*IL1RA*, *IL1B*	148 MS/ 98 HC	Higher EDSS progression associated with *IL-1RA* allele 2 + /*IL-1B* allele 2-	Candidate- gene (case–control) association study	*p* = 0.007	Not replicated	Schrijver HM. et al., 1999 [83]
Promoter variant (−1082)	*IL10*	116 MS/ 400 HC	Lower risk of severe disability (EDSS 6-8) for AG genotype	Candidate- gene (case–control) association study	*p* = 0.010; *p* = 0.026 (long-term) years	Not replicated	Luomala M. et al., 2003 [84]
Multiple SNPs	*CRYAB*	233 MS/ 96 HC	Trend toward slower EDSS progression for g.*CRYAB*-249 G/G genotype	Candidate- gene (case–control) association study	*p* = 0.07 (trend)	Not replicated	Stoevring B. et al., 2006 [36]
Multiple SNPs	*VDR*	512 MS (≥10-year follow-up)	Lower ff (30875) genotype frequency in MS with EDSS ≥ 6.0	Candidate- gene (longitudinal cohort) association study	OR = 0.38	Not replicated	Mamutse G. et al., 2008 [85]
Intergenic: rs6552511, rs7221818; *XYLT1*: rs12927173, rs2059283; MGAT: rs4953911, rs3814022	Intergenic regions; *XYLT1*; *MGAT5*	1040 MS (discovery) ** + 873 MS (replication)	Association with MSSS (*MGAT5*: lower MSSS), strongest in intergenic regions; replicated variants within *MGAT5*	GWAS (multi-cohort, with replication)	p (intergenic) ~10^−6^; p (*XYLT1*, *MGAT5*) ~10^−5^; p combined (discovery + replication for *MGAT5*) = 2.8× 10^−6^ and 1.5× 10^−7^	Partially replicated	Brynedal B. et al., 2010 [86]
M235T polymorphism	Angiotensinogen, angiotensin converting enzyme	195 MS/ 126 HC	Higher MSSS in TT homozygotes	Candidate- gene (case–control) association study	*p* = 0.02	Not replicated	Hladikova M. et al., 2011 [87]
C282Y, H63D	*HFE*	373 MS/ 129 HC	Worse disability (EDSS/MSSS) in C282Y	Candidate- gene (case–control) association study	*p* = 0.030	Not replicated	Bettencourt A. et al., 2011 [88]
5 SNPs	4 iron homeostasis genes (*HFE*, *FPN*, *HEPC*, *TF)*	414 MS/ 414 HC	Higher EDSS, MSSS, and PI in homozygous carriers of specific variants	Candidate- gene (case–control) association study	*p* = EDSS/MSSS/PI (HEPC: *p* = 0.003/0.001/-; FPN 0.01/0.01/-; HFE: -/-/0.009)	Not replicated	Gemmati D. et al., 2012 [89]
Combined genotype	HLA-DRB1*15:01 + CD24 (*v*/*v*) genotype	120 MS/ 120 HC	Higher MSSS in combined genotype carriers versus HLA-DRB1*15:01/x or -*CD24 v*/*v*	Candidate- gene (case–control) association study	*p* = 0.047	Not replicated	Ghlichnia HA. et al., 2014 [81]
rs6897932	*IL7RA*	270 MS/ 303 HC	Higher MSSS (MSSS > 6) associated with CC genotype	Candidate- gene (case–control) association study	*p* = 0.034	Not replicated	Cierny D. et al., 2015 [90]
rs9897526, rs5848	*GRN*	400 MS	Higher MSSS (>5) associated with rs9897526 A and rs5848 T alleles	Candidate- gene association study	*p* = 0.002; *p* = 0.0019	Not replicated	Vercellino M. et al., 2016 [60]
rs1800693, rs2104286 and rs6897932	*TNFRSF1A, IL2RA* and *IL7RA*	508 MS	Association with MSSS: *TNFRSF1A* rs1800693 T/T with milder disease (MSSS ≤ 3), *TNFRSF1AC* with more severe disease (MSSS >3)	Candidate- gene association study	*p* = 0.018	Not replicated	Kulakova OG. et al., 2016 [50]
rs12959006	*MBP*	127 first demyelinating event (5-year follow-up)	Higher annualized EDSS progression (ΔEDSS) in C/T + T/T genotype carriers	Candidate- gen association study (longitudinal)	*p* = 0.004	Not replicated	Zhou Y. et al., 2017 [91]
147 SNP panel	9 genes * in lymphocyte adhesion and trafficking (*ITGA4, SPP1*, etc.)	389 MS/ 336 HC	Higher MSSS associated with rs6721763 and rs6532040 (*ITGA4*, *SPP1*)	Candidate- gene (SNP panel, case–control) association study	*p* = 3.00 × 10^−6^; *p* = 0.009884	Not replicated	Dardiotis E. et al., 2017 [42]
38 SNPs **	21 genes (*PSMG4* *NLRP5* *MC1R*, etc.)	100 (discovery) + 2016 MS (replication)	Higher EDSS associated with *PSMG4* p.W99R and *NLRP5* p.M459I	Exome sequencing study (genome-wide, discovery) + targeted variant replication)	*p* = 0.002, 0.008	Nominal replication (targeted)	Sadovnick AD. et al., 2017 [65]
rs5844572 and rs755622	*MIF*	230 MS/ 248 HC	Higher EDSS and MSSS in male carriers	Candidate- gene (case–control) association study	*p* = 0.024; *p* = 0.034	Not replicated	Castañeda-Moreno VA. et al., 2018 [92]
rs10766197	*CYP2R1*	105 MS/ 130 HC	Faster disability progression (EDSS/MSSS) associated with A allele	Candidate- gene (case–control) association study	*p* < 0.05	Not replicated	Scazzone C. et al., 2018 [93]
3 SNPs: rs1800795, rs6897932, rs3212227	*IL6, IL7RA, IL12B*	297 MS	Higher MSSS associated with *IL-12B* rs3212227 C/C genotype in females	Candidate- gene association study	*p* = 0.04	Not replicated	Benešová Y. et al. 2018 [94]
rs3087456 and rs4774	*CIITA*	117 MS	Lower MSSS associated with rs3087456 A/A and rs4774 G/G	Candidate- gene association study	*p* < 0.0001	Not replicated	Pereira VCSR. et al., 2019 [95]
GOF variants: rs35829419, rs16944, rs479333	*NLRP3* *IL1B*	209 MS; 55 NMOSD	Severe disease (EDSS >5) and rapid progression (PI > 0.2)	Candidate- gene association study	*p* = 0.011; *p* = 6.6 × 10^−4^; *p* = 0.015	Not replicated	Soares JL. et al., 2019 [96]
rs244072 (C)	*ADA*	561 MS	Higher EDSS at diagnosis	Candidate- gene association study	*p* = 0.011	Not replicated	Bassi M. et al., 2020 [97]
rs948854 (G)	Galanin	110 MS	Higher MSSS (> 5) in minor allele carriers	Candidate- gene association study	*p* = 0.004	Not replicated	Luoudyno V. et al., 2020 [43]
rs7799039, rs1137101, rs8192678	*LEP*, *LEPR*, *PGC1A*	528 MS	Higher MSSS in male *LEPR* variant carriers	Candidate- gene association study	*p* = 0.005	Not replicated	Kolić I. et al., 2021 [98]
rs2821557 (C)	*KCNA3*	101 MS	Rapid disease course (MSSS > 7.5)	Candidate- gene association study	*p* = 0.027	Not replicated	Lioudyno V et al., 2021 [17]
rs9402373, rs9399005, rs12526196	*CTGF/CCN2*	200 RRMS/ 305 HC	Higher MSSS (≥2.4) in rs9399005 T/T and C/T genotypes	Candidate- gene (case–control) association study	*p* = 0.003	Not replicated	Can Demirdöğen B. et al., 2022 [99]
5 SNPs: rs1718119, rs2230911, rs2230912, rs3751143, and rs28360457	*P2* *X* *7 receptor*	128 MS/ 189 HC	Higher MSSS associated with GOF SNPs (rs1718119-A, rs22390912-G)	Candidate- gene (case–control) association study	*p* = 0.001	Not replicated	Guerini FR. et al., 2022 [100]
11 SNPs (thrombosis/coagulation-related polymorphisms)	Coagulation pathway (Factor V; Factor V-R2; HPA-1)	48 MS/ 25 HC	Higher disability and disease progression associated with *HPA-1a/1b* carriers	Candidate- gene (case–control) association study	*p* = 0.03 (disability); *p* = 0.02 (disease worsening); *p* = 0.01 (male subgroup)	Not replicated	Hadjiagapiou MS. et al. 2022 [101]

* GWAS on MS severity using data on 1040 MS patients; the two most significant SNPs (rs6552511 and rs7221818) were located in desert regions; few genes were represented by several SNPs (*MGAT, XYLT1, HIF1AN*). *XYLT1* and *MGAT5* were selected because they were represented by several SNPs in linkage disequilibrium. ** The primary analysis of the discovery cohort nominated 38 variants in 21 genes; a candidate-gene approach for the replication cohort was done.

## Data Availability

No new data were created or analyzed in this study. Data sharing is not applicable to this article.

## Abbreviations

The following abbreviations are used in this manuscript:

AAO	Age at onset
ABCB1	ATP-binding cassette subfamily B member 1 gene
AOMS	Adult-onset multiple sclerosis
APOE	Apolipoprotein E
ARR	Annualized relapse rate
ADA	Adenosine deaminase enzyme
BAEPs	Brainstem auditory evoked potentials
BDNF	Brain-Derived Neurotrophic Factor
BICAMS	Brief International Cognitive Assessment for Multiple Sclerosis
BRB	Rao’s Brief Repeatable Battery
CCR5	Chemokine receptor 5 gene
CI	Cognitive impairment
CIITA	Class II Major Histocompatibility Complex Transactivator
CPXM2	Carboxypeptidase X, M14 family, member 2
CRYAB	AlphaB-crystallin gene
CSF	Cerebrospinal fluid
CNS	Central Nervous System
CTGF/CCN2	Connective tissue growth factor gene
CTLA4	Cytotoxic T-lymphocyte antigen-4
DRD3	Dopaminergic receptor D3
DMTs	Disease-modifying treatments
DYSF ZNF638	Dysferlin-zinc finger protein 638
EBV	Epstein–Barr Virus
EDSS	Expanded Disability Status Scale
FN1	Fibronectin-1
FPN	Ferroportin
GFAP	Glial Fibrillary Acidic Protein
GM	Grey matter
GOF	Gain-of-function
GRN	Progranulin
GRS	Genetic risk score
GCIPL	Ganglion cell/inner plexiform layer
GWASs	Genome-wide association studies
HCs	Healthy controls
HEPC	Hepcidin
HLA	Human leukocyte antigen
HLA-GB	HLA genetic burden
HFE	Hemochromatosis
HPA1	Human platelet antigen 1;
ICAM-1	Intercellular adhesion molecule 1
IgG	Immunoglobulin G
IGSF9B	Immunoglobulin superfamily member 9B
IL	Interleukin
IL1-beta	Interleukin-1-beta
IL1R-alpha	Interleukin-1 receptor alpha
IL7RA	Interleukin 7 receptor A
IMSGC	International Multiple Sclerosis Genetics Consortium
KCNA3	Potassium Voltage-Gated Channel Subfamily A Member 3
KFLCs	Kappa Free Light Chains
LEP	Leptin
LEPR	Leptin receptor
LRP2	Low-density lipoprotein receptor-related protein 2
LOMS	Late-onset multiple sclerosis
MBP	Myelin basic protein
MC1R	Melanocortin-1 receptor
MGAT5	Alpha-1,6-Mannosylglycoprotein 6-Beta-N-Acetylglucosaminyltransferase
MHC	Major histocompatibility complex
MIF	Macrophage migration inhibitory factor
MRI	Magnetic resonance imaging
MS	Multiple sclerosis/Patient with multiple sclerosis
MSFC	Multiple Sclerosis Functional Composite
MSSS	Multiple sclerosis severity score
MTHFR	Methylenetetrahydrofolate reductase
NAT1	N-acetyltransferase-1
NEDA-3	No evidence of disease activity-3
NGS	Next-generation sequencing
NfLs	Neurofilament light chains
NLRP5	NLR Family Pyrin Domain Containing 5
OCBs	Oligoclonal bands
OCT	Optic Coherence Tomography
OPN	Osteopontin gene
OR	Odds ratio
PGC1A	Peroxisome proliferator-activated receptor gamma co-activator 1-alpha
PI	Progression index
PPAR-γ	PPAR-gamma
PMS	Progressive multiple sclerosis
POMS	Pediatric-onset multiple sclerosis
PPMS	Primary-progressive multiple sclerosis
PRS	Poligenic Risk Score
PSMG4	Proteasome Assembly Chaperone 4
RNFL	Retinal nerve fiber layer
RRMS	Relapsing–remitting multiple sclerosis
SDMT	Symbol Digit Modalities Test
SIRT1	Sirtuitin 1
SNPs	Single nucleotide polymorphisms
SPMS	Secondary-progressive multiple sclerosis
SSEPs	Somatosensory evoked potentials
TF	Transferrin
TNF-alpha	Tumor necrosis factor-alpha
TNFRSF1A	TNF receptor superfamily member 1A
TRAIL	TNF-related apoptosis-inducing ligand
VDR	Vitamin D receptor
VEPs	Visual evoked potentials
WNT9B	Wnt family member 9B
